# Death from Chloroform

**Published:** 1856-06

**Authors:** 


### DEATH FROM CHLOROFORM.

We have to record another death from the inhalation of chloroform. It occurred in Boston. The exhibitor, Dr. Emery, publishes the following statement of the case in the *Boston Journal:*

"Between the hours of 1 and 2 o'clock, on the 5th inst., I commenced to administer chloroform to Mrs. P. A. Morgan, at her request, for the purpose of removing some teeth. I commenced with a small quantity—should think from two to three drachms, on a sponge. She inhaled it with difficulty for a minute or two. Her pulse was not strong, but uniform. She then commenced to be excited, and said that I was going to extract her teeth, and she should know all about it. She said that Mrs. Paige (the lady who accompanied her) was getting the forceps to extract them with. I think about one minute had passed during this conversation and excitement. I then removed the sponge from her mouth, and in a few moments she became quiet, and satisfied that there had been no attempt to remove her teeth. In a few moments I commenced the operation again, with the same amount of chloroform. She inhaled it without difficulty about as long as she did before, and became so much excited that she got up out of the chair, and insisted that I had extracted her teeth. She spit on the floor and looked to see if it was blood, and she insisted that some one was coming into the room whom she did not wish to see. I sat her down in the chair again, and she then went into a spasm, closed her teeth and breathed with difficulty. I sprinkled water on her face, and the muscles relaxed, and I asked her to get up and we would place her on the lounge. She made an effort to rise, and, with my assistance, stood on her feet, and then instantly sank to the floor. With the assistance of Mrs. Paige, I placed her on the lounge, and then there was a rush of blood to the brain. I sprinkled water in her face again, but she showed no signs of being conscious. Mrs. Paige went for assistance, and I immediately commenced artificial respiration by insufflation, and kept it up until Dr. Stedman came in, which was but a few minutes."—*Medical News.*

